# Hydroxytyrosol-Fortified Foods Obtained by Supercritical Fluid Extraction of Olive Oil

**DOI:** 10.3390/antiox10101619

**Published:** 2021-10-14

**Authors:** Lucia Bartella, Fabio Mazzotti, Ines Rosita Talarico, Ilaria Santoro, Leonardo Di Donna

**Affiliations:** 1 Dipartimento di Chimica e Tecnologie Chimiche, Università della Calabria, Via P. Bucci, Cubo 12/D, I-87030 Rende, CS, Italy; lucia.bartella@unical.it (L.B.); fmazzotti@unical.it (F.M.); inesrosita.talarico@unical.it (I.R.T.); 2 QUASIORA Laboratory, Agrinfra Research Net, Università della Calabria, Via P. Bucci, Cubo 12/D, I-87036 Arcavacata di Rende, CS, Italy; ilariasantoro@yahoo.it

**Keywords:** hydroxytyrosol, functional foods, CO_2_ supercritical fluid extraction, flour, whole-wheat flour, sugar

## Abstract

A new original process has been set-up to obtain hydroxytyrosol-enriched food by means of CO_2_ supercritical fluid extraction. The process is based on the direct adsorption of hydroxytyrosol on selected foodstuff (e.g., flour, whole-wheat flour, and sugar) when placed in contact with olive oil (which is known to contain this phenol) under controlled temperature and pressure conditions of supercritical CO_2_. The transfer of the nutraceutical to the foodstuff was initially evaluated using a fortified olive oil model and was then applied to commercial olive oil and foodstuff. The yield of the hydroxytyrosol transfer was demonstrated to be quantitative. In order to prove the suitability of the final products, hydroxytyrosol-enriched flour was used to prepare a bread roll, which maintained the nutraceutical characteristics after the cooking. Finally, DPPH based experiments were performed to prove the radical scavenging activity of the functionalized foodstuff.

## 1. Introduction

A functional food is a product whose consumption is associated with health improvements with respect to its ordinary nutritional properties. Its main aim is to reduce disease risks through prevention [1]. As reported by the Food and Agricultural Organization of the United Nations [2], a functional food should resemble, in appearance, a conventional food, and, in addition, should have physiological benefits through the presence of biologically active components. In general, a functionalized food is obtained by adding to the traditional foodstuff a natural or synthetic extract containing active principles such as vitamins, antioxidants, bioactive peptides, probiotics, or, simply, oligoelements [3]. There are many examples of functionalized food present on the market or yet at a proof of concept level; for example, fish oils containing omega-3 fatty acids are considered a functional food that helps to lower the blood cholesterol level; lactobacillus bacteria added to yogurt may contribute to improve the quality of intestinal microflora; and olive oil for its content of vitamin E and phenols helps to protect tissues from oxidative stress [4]. The market of functional foods is constantly growing, and, globally, is expected to reach a business volume of 255 billion dollars by 2024 [5]. Recent international regulations [6] allow health beneficial effects to be highlighted directly on the label of the food, giving an opportunity to boost the market of functionalized foods. As an example, according to the latter regulation, the olive oil phenols (hydroxytyrosol-based compounds) may “contribute to the protection of blood lipids from oxidative stress” only if the concentration of these active molecules are above 5 mg per 20 g of olive oil; hence, the olive oils rich in those phenols become a full-fledged functional food. It has been demonstrated that hydroxytyrosol possesses several beneficial effects on human health: numerous studies have shown its protection activity against metabolic diseases [7] and digestive disorders [8] and its anti-carcinogenic [9], anti-inflammatory [10], and antimicrobial activity [11]. Furthermore, hydroxytyrosol has no genotoxic and no mutagenic effects [12]; this enhances its potential as nutraceutical for functional foods. Furthermore, several studies have shown that the concentration of simple phenols, such as hydroxytyrosol, increases during the storage and the aging of olive oil because of the degradation of their derivatives. In particular, the degradation of oleuropein, its aglycone, and oleacein causes a simultaneous increase of hydroxytyrosol [13,14,15,16]. However, the aging of the olive oil causes important deteriorative changes in its chemical, sensory, and nutritional properties, affecting the shelf life of the olive oil, which is less than 24 months. Here we present a simple process, based on CO_2_ supercritical fluid extraction (SFE), which allows for the selective transfer of the nutraceutical hydroxytyrosol from olive oil to selected foodstuffs, aiming at the design of enriched foods with radical scavenging activity. This work may open new perspectives for the re-use of aged olive oils, giving, at the same time, the opportunity to develop a new generation of functional foods.

## 2. Materials and Methods

### 2.1. Food Samples

Flour (white bread flour from wheat), whole-wheat flour, sugar (regular fine sugar from beets), and extra virgin olive oil were purchased at a local market (Cosenza, Italy).

### 2.2. Chemicals and Standards

Analytical grade methanol, ethanol, acetonitrile, formic acid, and DMSO were supplied by VWR International (Radnor, PA, USA). Ultrapure water was obtained from a Milli-Q plus system (Millipore, Bedford, MA, USA). Hydroxytyrosol was purchased from Extrasynthese (Genay Cedex, France) and d_2_-hydroxytyrosol was obtained in our laboratory following a method from the literature [17].

### 2.3. Fortified Samples Preparation-Supercritical Fluid CO_2_ Extraction (SFE)

Twenty-four grams of each food sample were mixed with 6 g of olive oil using an agate mortar for a few minutes to allow a homogeneous dispersion of oil in the food. The mixture was extracted by supercritical fluid using carbon dioxide as the extraction solvent. The extraction was carried out by a Spe-ed SFE 2 system (Applied Separation, Allentown, PA, USA) composed of a CO_2_ tank, a pump to pressurize the gas, an oven with two extractor vessels, a restrictor to maintain a high pressure in the extraction system, and two metrical valves. Thirty grams of the mixture was placed in a 50 mL extractor vessel. Supercritical extractions were performed in combined mode, consisting of a step of 60 min in the closed system at 40 °C, 100 bar, and a subsequent step accomplished at the open system at 40 °C, 300 bar, to allow for the complete removal of the oil components. The supercritical CO_2_ flow was set at 5 mL/min and the restrictor temperature was set at 120 °C. Both the enriched foodstuff and the extracted oil were collected and analyzed by HPLC-UV/MS (Waters, Milford, MA, USA). The best conditions of temperature and pressure of the SFE process were chosen by the results of the HPLC-UV and HPLC-MS/MS analyses performed on the fortified food, evaluating the maximum yield of the transfer of hydroxytyrosol in the food.

### 2.4. Instrumental Analysis

#### 2.4.1. Sample Preparation for HPLC-UV/MS Analysis

One gram of the oil sample was extracted with 10 mL of MeOH at room temperature. After centrifugation, the supernatant was submitted to a defatting step with n-hexane to completely remove the lipid fraction: briefly, the extract was washed with 10 mL of hexane three times. The methanolic extract was concentrated under vacuum at 35 °C and then re-dissolved in 300 µL of MeOH and finally analyzed by HPLC-UV/MS.

For food matrices, 4 g of each sample were extracted at room temperature with 20 mL of MeOH. The mixture was then stirred by vortex for 5 min and then centrifuged at 2500× *g* for 5 min. The methanolic extract was quantitively recovered and directly analyzed.

#### 2.4.2. Sample Preparation for Mass Spectrometric Analysis

For the olive oil, the oil samples were treated according to the procedure present in the literature [18,19]: 25 µL of a d_2_-hydroxytyrosol solution at 2000 mg/L was mixed to 2 g of sample. The resulting mixture was homogenized by ultra-turrax (IKA Works, Inc., Staufen, Germany) for 3 min, and then 1 g was extracted using a 1 mL solution of 0.1% HCOOH/EtOH 3:7 *v*/*v*. After the extraction, the centrifuged supernatant was diluted (1/10) in 1 mL of MeOH and injected.

For the flour, whole-wheat flour, sugar, and bread, 1 g of sample was added to twenty microlitres of d_2_-hydroxytyrosol solution at 50 mg/L, mixed by vortex for 3 min, and finally extracted with 10 mL of MeOH. The supernatant was quantitatively recovered after centrifugation and directly injected. 

#### 2.4.3. HPLC-UV/MS

HPLC-ESI-UV/MS analysis was performed using a FractionLynx system from Waters (Milford, MA, USA) working in analytical mode, equipped with an Acquity QDa mass spectrometer and a 2489 UV/visible detector. The chromatographic separation was carried out using a C_18_ reversed-phase column, Luna (250 × 4.6 mm, 5 μm, Phenomenex, Torrance, CA, USA), at a flow rate of 1 mL/min, injecting a volume of 20 µL. The run time was 70 min, and the gradient was built using 0.1% HCOOH in H_2_O (solvent A) and MeOH (solvent B) as mobile phases. The elution gradient comprised the following steps: isocratic elution, 95% A for 7 min—linear gradient from 95% A to 60% A in 33 min, linear gradient from 60% A to 40% A in 5 min, and linear gradient from 40% A to 10% A in 5 min; and isocratic elution, 10% A for 7 min—linear gradient from 10% A to 95% A in 5 min and equilibration of the column for 8 min. The UV detector was set at 280 nm.

#### 2.4.4. Mass Spectrometry

HPLC-MS/MS analysis was carried out on a system composed of a UHPLC Accela pump coupled to a TSQ Quantum Vantage triple-stage quadrupole mass spectrometer (Thermo Fisher Scientific, San José, CA, USA). The chromatographic analysis was performed with a C_18_ reversed-phase column, Hypersil (2.1 × 50 mm, 3 μm particle size, Thermo Fisher Scientific, San José, CA, USA). H_2_O (A) and CH_3_CN (B) were used as the solvents for chromatographic separation, and the elution gradients were as follows: at t = 0.0 min, 100% A; at t = 1.0 min, 100% A; at t = 6.0 min, 10% A and 90% B; at t = 8.0 min, 10% A and 90% B; at t = 9.0 min, 100% A; and at t = 12.0 min, 95% A and 5% B. The flow rate was set at 0.3 mL/min, and the sample injection volume was 10 µL. A further switching valve located on the mass spectrometer was utilized to divert the LC flow to waste at the first minute and after 4.70 min of each analysis to protect the MS source from contamination. Mass spectrometry was performed, acquiring spectral data on a triple-quadrupole mass analyzer equipped with a heated electrospray ionization (HESI II) source operating in negative ion mode with the following conditions: spray voltage, −3.5 kV; capillary and vaporizer temperatures, 270 °C and 280 °C, respectively; and auxiliary and sheath gas at 46 and 40 arbitrary units (au), respectively. Quantitative analysis was performed by multiple reaction monitoring (MRM) following two specific analyte transitions either for hydroxytyrosol or for d_2_-hydroxytyrosol: the first one was used for assay and the second for confirmation as reported elsewhere [19]. The collision induced argon pressure (CID) was set at 1.0 mTorr, and the mass resolution at the first (Q1) and third (Q3) quadrupoles was set at 0.7 Da at full width at half maximum (FWHM). The S-lens rf amplitude and the collision energy (CE) were optimized individually per compound. All valve positions and instrument parameters were controlled by Xcalibur software, version 2.0.0 (Thermo Fisher Scientific, San José, CA, USA). The total HPLC-MS/MS method run time was 12 min. 

### 2.5. Antioxidant Capacity Assays

#### 2.5.1. Sample Preparation 

Four grams of each food product was extracted with 20 mL of methanol. The resulting mixture was stirred by vortex for 5 min and then centrifuged at 2500× *g* for 5 min. The methanolic extract was filtered, concentrated using a rotary evaporator (Heidolph Instruments, Schwabach, Germany) at 35 °C, and dissolved in 1 mL of DMSO.

#### 2.5.2. DPPH Radical Scavenging Activity 

DPPH radical scavenging activity was determined spectrophotometrically at 517 nm by measuring the DPPH concentration after the reaction with the antioxidant molecules present in food extracts [20]. Briefly, 100 µL of extracted sample was mixed with 800 µL of ethanolic solution of DPPH radical (40 mg/L) in a cuvette. The DPPH absorbance was measured every 30 s for 30 min. Lower absorbance values of the reactive mixture indicate a higher free radical scavenging activity. The results of the DPPH experiments were expressed as percent of inhibition of DPPH solution, calculated as follows: I% = [(A_control_ − A_sample_)/A_control_] × 100, where A_control_ is the absorbance of 800 µL of DPPH solution with 100 µL of DMSO and A_sample_ is the absorbance of DPPH in the presence of food samples. Spectrophotometric analysis was carried out using a spectrophotometer Evolution 600 UV/VIS (Thermo Fisher Scientific, San José, CA, USA).

## 3. Results

Here we present a novel methodology to obtain new functional foods containing hydroxytyrosol, a nutraceutical component naturally found in olive oil, which is well known for its antioxidant properties. This methodology relies on the use of CO_2_ supercritical fluid extraction to fortify foodstuffs consumed daily in our diet. Generally, carbon dioxide, which becomes supercritical at temperatures and pressures above 31.2 °C and 72.9 atm, respectively, is used to eliminate unwanted molecules or classes of compounds from a foodstuff; this is the case for decaffeinated coffee, defatted potato chips, etc. At supercritical conditions, CO_2_ is compressible such as a gas but possesses a density and a solvating power similar to a liquid, having low viscosity and high diffusivity; it diffuses easily through solid materials and provides faster extraction yields. In this application, the extraction process is used to transfer a target polar compound from a fat matrix to another food. The procedure has been optimized to perform a quantitative transfer of hydroxytyrosol from olive oil to foodstuffs in the shortest possible time.

### Supercritical CO_2_ Fluid Extraction to Produce Fortified Foodstuff

**Figure 1:**
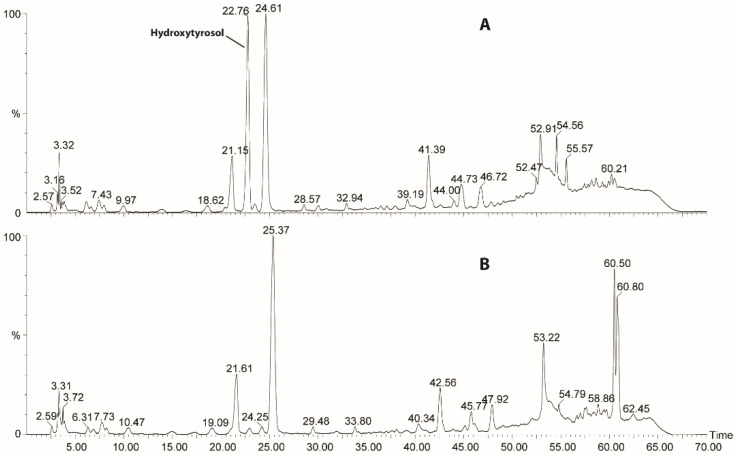
HPLC-UV chromatograms at λ = 280 of the methanolic extract of the flour (**A**) after the SFE experiments and (**B**) before the procedure.

We have prepared three different types of hydroxytyrosol-enriched food: flour, whole-wheat flour, and sugar. In general, the starting material (e.g., the original foodstuff) is mixed with olive oil to allow the maximum dispersion of the oil in the food matrix; then, the mixture is submitted to the supercritical fluid process to obtain the final hydroxytyrosol-enriched product after the elimination of the lipid component via the outlet valves of the system. In order to optimize the process, several experiments were conducted on the flour matrix using an olive oil sample spiked with standard hydroxytyrosol at a concentration of 100 mg/L. The experiments were carried out with varying supercritical process conditions, i.e., the temperature, the pressure, and the duration of experiment. It is known that the solubility of an analyte in a solvent is highly influenced by the density of the solvent, while the density of a fluid state is particularly sensitive to small changes in pressure and temperature [21,22]. The first set of experiments was performed at a fixed pressure of 400 bar, varying either the temperature (from 40 °C to 70 °C) or the time (from 1 to 3 h). It was observed that the increase of the temperature does not influence the transfer of hydroxytyrosol to the flour matrix. Therefore, the subsequent set of experiments was carried out at the lowest values of temperature and pressure, i.e., 40 °C and 100 bar, respectively, varying only the time from 1 h to 3 h. The results of these tests have shown that the quantitative transfer of hydroxytyrosol may be achieved in 3 h. Furthermore, in order to decrease the time of the process, other experiments were accomplished in a combined mode, which is a two-step experiment: in the first step the system works in static mode (closed system), at constant values of temperature and pressure (40 °C, 100 bar), while the second step, on the contrary, involves the dynamic operating mode (open system) in which the pressure is increased up to 300 bar. The best results were achieved with the following conditions: static mode at 40 °C and 100 bar for 1 h and dynamic mode at 40 °C and 300 bar for 30 min. After the experiments, the flour was recovered from the vessel and analyzed by HPLC-UV to evaluate the presence of hydroxytyrosol. Figure 1 reports the chromatograms of the methanol extract of the sample before and after the transfer process.

Figure 1A, which represents the sample after the SFE process, clearly demonstrates the presence of the hydroxytyrosol signal in the recovered flour. The quantitative assay, performed using the external standard method, confirmed the quantitative transfer of hydroxytyrosol from the oil to the flour.

**Figure 2:**
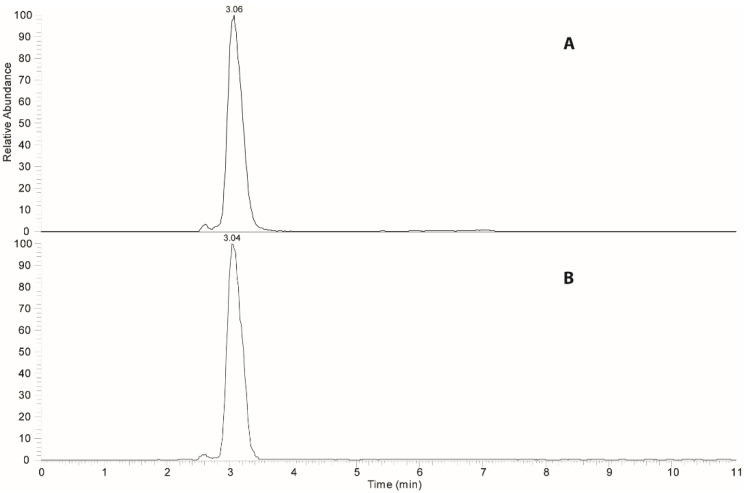
Multiple reaction monitoring chromatograms performed on a sample of enriched flour: (**A**) transition relative to hydroxytyrosol and (**B**) transition relative to *d*_2_-hydroxytytrosol (internal standard).

The process was then applied on selected foods using a commercial olive oil containing a concentration of 14 mg/kg of hydroxytyrosol; flour, whole-wheat flour, and sugar were chosen as sample target foods for the hydroxytyrosol transfer. At the conclusion of the transfer process, each sample was extracted and analyzed by HPLC-ESI-MS/MS under MRM conditions following the procedure already reported in the literature [18]. Figure 2 shows a representative MRM chromatogram of an enriched sample of the flour after the transfer process, revealing the signals due to the transitions of hydroxytyrosol and the labeled internal standard.

It is worth noting that the analyzed olive oil fraction recovered at the exit of the outer valve has been also analyzed by HPLC-ESI MS/MS in order to control the amount of hydroxytyrosol remaining in the fat. Figure S1 displays the absence of the signal relative to phenol, confirming the quantitative transfer to the foodstuff. Table 1 shows the amount of hydroxytyrosol found in enriched foods after the extraction process and the relative transfer yield values, which are near 100%, meaning hydroxytyrosol completely transferred from the olive oil to the food.

In order to be sure that the enriched food products could have an effective outcome in daily diets, a bread roll was prepared using the hydroxytyrosol-enriched flour. The roll was then cooked in the oven at 180 °C for 30 min.

**Figure 3:**
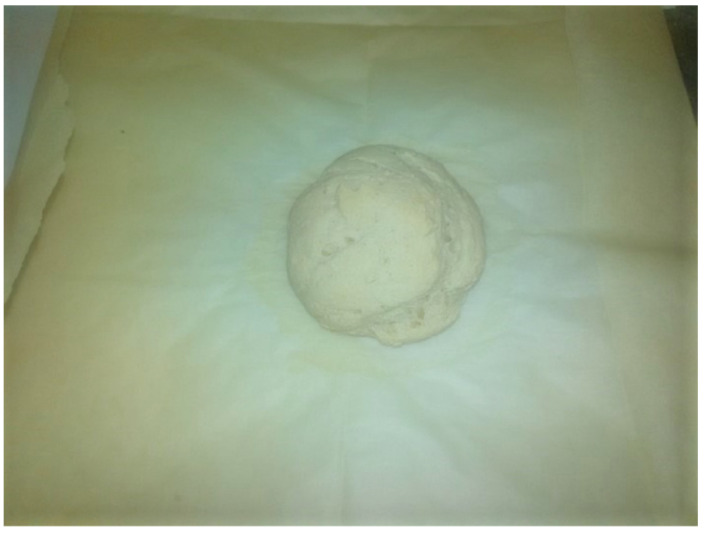
The bread roll obtained by enriched flour after cooking at 180 °C for 30 min.

At the end of the cooking process, both the rind and the inner part of the “enriched bread” (Figure 3) were extracted and analyzed by HPLC-MS/MS to verify the presence of hydroxytyrosol.

Table S1 shows the amount of hydroxytyrosol in the cooked bread obtained from the enriched flour, highlighting that no degradation of hydroxytyrosol occured.

Finally, the ability of the hydroxytyrosol-enriched novel foods to scavenge free radicals was evaluated. The antioxidant capacity of enriched foods was determined by DPPH assay. This test involved the measurement of DPPH radical absorbance at 517 nm during the reaction with antioxidant compounds present in foods. A DPPH radical is an organic nitrogen radical with a deep purple color, and when mixed with an antioxidant/reducing compound its color changes from purple to yellow, which indicates the formation of hydrazine. 

**Figure 4:**
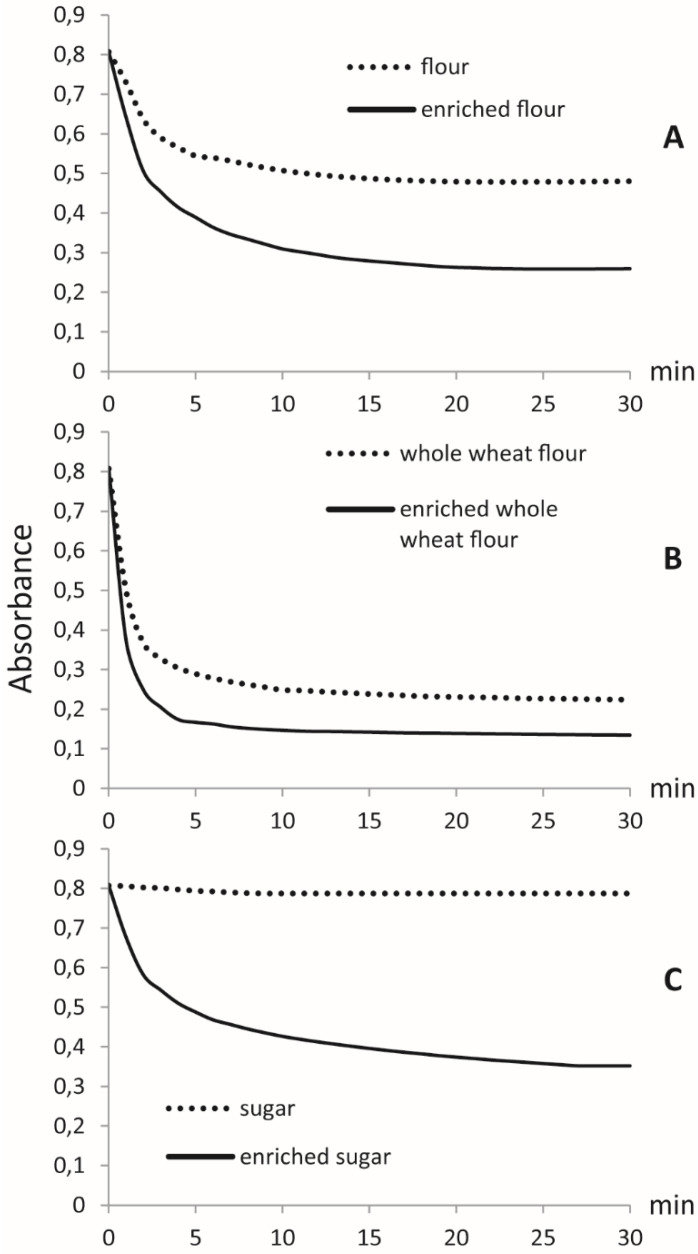
The kinetic curves obtained by the scavenging activity of DPPH performed on foods. (**A**) flour vs. enriched flour. (**B**) whole-wheat flour vs. enriched whole-wheat flour. (**C**) sugar vs. enriched sugar.

For each food sample, two independent assays were performed: the first on original matrices and the second on enriched foods. The experiments were performed with an excess of DPPH in order to run out the H-donating capacity of the antioxidant compounds. The spectrophotometric measurements were performed for 30 min until the reaction reached the equilibrium. The kinetic curves were obtained by plotting the DPPH radical absorbance versus the time of experiment (Figure 4).

The kinetic behavior showed a lower DPPH absorbance value for fortified foods at the end of the reaction compared to the original products. In particular, the kinetic curve shows that the disappearance of the DPPH radical is faster in the fortified flour than in the original food sample, and the same effect is observed for the whole-wheat flour sample. For sugar, the absorbance difference between the two samples is much higher, because the original sugar matrix does not show any radical scavenging activity (Figure 4). For all experiments, the reaction with DPPH was biphasic [20], with a fast decay in absorbance in the first few minutes and a slower step in which the equilibrium was reached, when the DPPH reagent was degraded. The results were expressed in terms of reduction percentage of the DPPH solution or percent of inhibition, through the absorbance values at the beginning and after 30 min (Table 2), showing a much higher radical scavenging activity for fortified foods.

## 4. Discussion

The results reported above demonstrate the possibility of employing supercritical CO_2_ fluid extraction to easily enrich common foods with hydroxytyrosol, which is known to possess important antioxidant properties acknowledged by international regulations. Regarding the latter point, the European Union, through the regulation 432/2012 [6], allows a sentence on the label of olive oils rich in hydroxytyrosol that reports on the protection exerted by this nutraceutical against oxidative stress on blood lipids. The above reported results quantitatively show the transfer of hydroxytyrosol using supercritical CO_2_ as a carrier. In the process, the supercritical CO_2_ extraction is used in an unconventional way; in fact, this type of extraction, known for its sustainability in terms of the environment, usually removes undesired components from foodstuff (an example is given by the large-scale production of decaffeinated coffee). In this work, on the contrary, the supercritical CO_2_ works as a medium transfer of hydroxytyrosol from the olive oil to a solid food such as flour or sugar. The transfer is made possible because of the polar nature of hydroxytyrosol, which is preferentially adsorbed on the polar components of the solid foodstuff. This hypothesis has been proved by analyzing the exhausted oil transported out of the column by the CO_2_ flow; in fact, the oil shows no evidence of the polar phenol (Figure S1), which, on the contrary, is retained on the surface of the solid (Figure 1). A further finding regards the amount of oil recovered from the exit valve of the SFE apparatus after the extraction process: no trace of olive oil or lipid material remains in the functionalized foods, leaving unchanged the aspect and the properties of the original food. Furthermore, the antioxidant capacity of the enriched foodstuff has been demonstrated by chemical tests using DPPH. Figure 4 clearly shows that this radical works in a more effective manner on the food obtained after the transfer procedure, rather than on the original one. This means that the presence of hydroxytyrosol adds further antioxidant activity to the enriched foods. In addition, the results obtained have highlighted that the transfer of hydroxytyrosol from olive oil to food matrices is quantitative, and the new product can realistically be used in daily diets because the hydroxytyrosol does not suffer any degradation under cooking conditions. The quantitative transfer is an important feature of the process: in fact, it would be useful to use aged olive oils as the main ingredient, as they are known to contain higher concentrations of free hydroxytyrosol produced by degradation processes. This new approach based on the transfer of molecules under supercritical CO_2_ may be extended to other binary matrixes in order to create functional food enriched with different nutraceuticals.

## 5. Conclusions

An original method describing the formulation of innovative functionalized foods containing the antioxidant molecule hydroxytyrosol has been developed. The method relies on the CO_2_ supercritical fluid extraction of a mixture containing common foods such as wheat, flour, whole wheat flour, or white sugar and olive oil. The method, based on a two-step process (static mode and dynamic mode), accomplishes a quantitative transfer of hydroxytyrosol from olive oil to foods, leaving their characteristics unaltered. DPPH tests were performed on the enriched foods, showing an increase of their antioxidant activity. Finally, the flour was used to prepare a bread roll which maintained the presence of the nutraceutical after the cooking process.

## Figures and Tables

**Table 1:** Amount of hydroxytyrosol transferred to food from a commercial olive oil and transfer yields.

	Amount of Hydroxytyrosol (µg)	Transfer Yield %
Olive oil	82.32	n/a *
Flour	82.24	99.90
Whole-wheat flour	81.20	98.64
Sugar	81.92	99.51

* n/a not applicable.

**Table 2:** Percent of inhibition of enriched foodstuffs compared to original samples.

Sample	% Inhibition
Flour Enriched flour	38.80 64.38
Whole wheat flour Enriched whole wheat flour	74.65 86.11
Sugar Enriched sugar	2.55 58.28

## Data Availability

Data is contained within the article or Supplementary Materials.

## Supplementary Materials

The following are available online at https://www.mdpi.com/article/10.3390/antiox10101619/s1: Figure S1. Multiple reaction monitoring chromatograms performed on the exhausted olive oil: (A) transition relative to hydroxytyrosol, (B) transition relative to *d*_2_-hydroxytytrosol (internal standard); Table S1. Total amount of hydroxytyrosol found in “enriched bread roll”.
