# Supplementary material for: Hydroxytyrosol-Fortified Foods Obtained by Supercritical Fluid Extraction of Olive Oil
Source: Antioxidants (Basel). 2021 Oct 14;10(10):1619. doi: 10.3390/antiox10101619 (PMC8533198; doi:10.3390/antiox10101619)
Supplement: Supplementary file 1 [file antioxidants-10-01619-s001.zip › antioxidants-1405503-supplementary.pdf]

Hydroxytyrosol enriched-foods obtained by supercritical fluid extraction of olive oil

Supplementary material

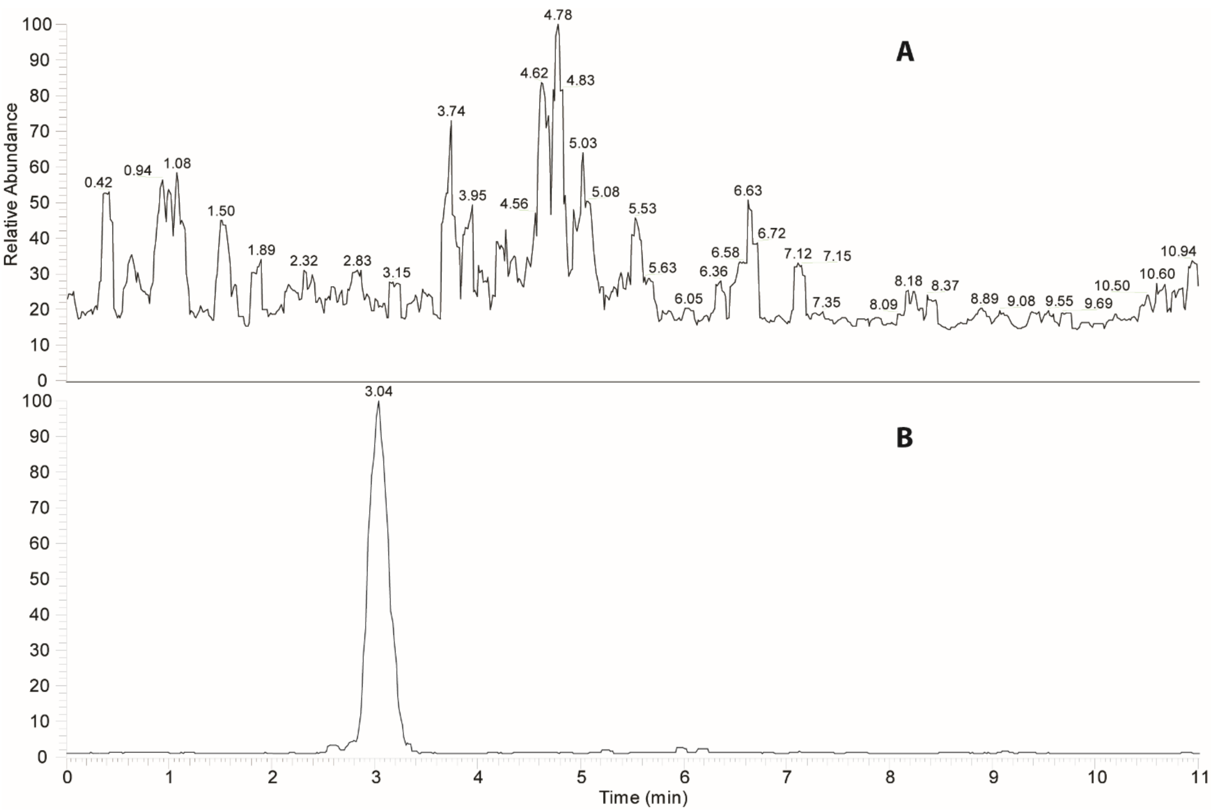

Figure S1. Multiple reaction monitoring chromatograms performed on the exhausted olive oil: (**A**) transition relative to hydroxytyrosol, (**B**) transition relative to d_2-hydroxytytrosol (internal standard)

Table S1. Total amount of hydroxytyrosol found in "enriched bread roll"

**Amount of hydroxytyrosol in enriched flour (μg)**	69.20
**Amount of hydroxytyrosol in bread rind (μg)**	16.61
**Amount of hydroxytyrosol in bread crumb (μg)**	49.31
